# Rigidity of loop 1 contributes to equipotency of globular and ribbon isomers of α-conotoxin AusIA

**DOI:** 10.1038/s41598-021-01277-4

**Published:** 2021-11-09

**Authors:** Thao N. T. Ho, Nikita Abraham, Richard J. Lewis

**Affiliations:** Institute for Molecular Bioscience, The University of Queensland, Brisbane, 4067 Australia

**Keywords:** Structural biology, X-ray crystallography, Peptides, Membrane proteins

## Abstract

α-Conotoxins are small disulfide-rich peptides targeting nicotinic acetylcholine receptors (nAChRs) characterised by a C^I^C^II^-X_m_-C^III^-X_n_-C^IV^ framework that invariably adopt the native globular conformations which is typically most potent. α-Conotoxins are divided into several structural subgroups based on the number of residues within the two loops braced by the disulfide bonds (m/n), with the 4/7 and 4/3 subgroups dominating. AusIA is a relatively rare α5/5-conotoxin isolated from the venom of *Conus australis.* Surprisingly, the ribbon isomer displayed equipotency to the wild-type globular AusIA at human α7-containing nAChR. To understand the molecular basis for equipotency, we determined the co-crystal structures of both isomers at *Lymnea stagnalis* acetylcholine binding protein. The additional residue in the first loop of AusIA was found to be a critical determinant of equipotency, with 11-fold and 86-fold shifts in potency in favour of globular AusIA over ribbon AusIA observed following deletion of Ala4 or Arg5, respectively. This divergence in the potency between globular AusIA and ribbon AusIA was further enhanced upon truncation of the non-conserved Val at the C-termini. Conversely, equipotency could be replicated in LsIA and TxIA [A10L] following insertion of an Ala in the first loop. These findings provide a new understanding of the role the first loop in ribbon and globular α-conotoxins can play in directing α-conotoxin nAChR pharmacology.

## Introduction

nAChRs are prototypical members of the ligand-gated ion channel class of membrane proteins that are found extensively in the central (CNS) and peripheral nervous system (PNS). nAChRs are interesting therapeutic drug targets associated with the progression of CNS disorders such as epilepsy, Alzheimer’s disease, Parkinson’s disease, and schizophrenia^1–3^. Neuronal nAChRs are assembled as homopentamers of α7, α8 and α9 or heteropentamers of α2–α6 in complex with β2–β4 or α7 with β2 subunits or α9 with α10 subunits (α8 subunit only reported in avian libraries^4^). Ligand binding in nAChRs is at the interface between two adjacent subunits, in which one subunit contributes to the principal face and the other contributes to the complementary face of the ligand binding site^5,6^. Generally, in heteromeric nAChRs, the principal face comes from one α subunit, while the complementary face arises from non-α subunit, except for heteropentameric α9α10 nAChRs. Recently, some nAChRs were also found to be expressed in non-neuronal cells, such as α7 nAChRs in immune system, muscles, skin, lung^7^, or α3β4 nAChRs in lung cancer cells^8^, or α9α10 nAChRs in auditory hair cells, cancer cells and immune cells^9^. Upon ligand binding, the five subunits move relative to each other as nAChRs undergo conformational changes between functional states^10^. The structural features of the ligand binding site, which is unique for different subunits, and the specific amino acid interactions between ligands determine the pharmacological properties of different nAChR subtypes.

Venoms from cone snails termed conotoxins comprise a cocktail of toxins, mostly small disulfide-bridged peptides, with high affinity and precise selectivity for different types of receptors and ion channels such as voltage-gated and ligand-gated ion channels as well as G-protein-coupled receptors and transporters. Conotoxins are grouped into several “superfamilies” and “families”, which include the enhancers of sodium channels (δ-conotoxin), blockers of sodium, calcium, potassium channels (μ-, ω-, κA-conotoxin, respectively), as well as competitive and non-competitive antagonists of nAChRs (α- and ψ-conotoxin, respectively)^11,12^. α-Conotoxins are among the smallest conopeptides (12–20 amino acids (aa)) from *Conus* venoms, with most acting as competitive antagonists of nAChRs. Classical α-conotoxins are characterised by a C^I^C^II^-X_m_-C^III^-X_n_-C^IV^ framework forming three possible disulfide connectivities: globular (I–III, II–IV), ribbon (I–IV, II–III) and bead (I–II, III–IV)^13–15^. The globular conformation is generally the native and most potent isomer, while the ribbon and bead isomer typically show weak or no inhibition. α-Conotoxins are further divided into different structural subgroups (*m/n*: 3/5, 5/5, 4/3, 4/4, 4/5, 4/6 and the common 4/7) based on the number of residues within the two loops (*m*, *n)* braced by the disulfide bonds. Target selectivity also roughly correlates to the loop size, with the 3/5 framework active at neuromuscular nAChRs, while the 5/5, 4/3, 4/4, 4/5, 4/6 and 4/7 subgroups mainly active at neuronal nAChRs. Despite the remarkable selective for different subtypes of nAChRs as well as the peptides’ outstanding stability in biological system making conotoxins interesting drug candidates, the inability to cross the blood–brain barrier due to its polar nature hinders further its development into CNS drug candidates^16^.

AusIA isolated from the venom of *C. australis* is the first α-conotoxin characterized as an α5/5-conotoxin where both loops (m/n) comprise of 5 amino acids^17^. To better understand the structure–function of this group, both globular and ribbon conformation of AusIA (gAusIA and rAusIA, respectively) were synthesized and characterised. Interestingly, in contrast to other α-conotoxins investigated, gAusIA and rAusIA were equipotent at chicken α7 nAChRs heterologously expressed in *Xenopus laevis* oocytes^17^. NMR revealed both isomers lacked a uniquely folded α-helical structure, although rAusIA appeared more stable due to the presence of one major conformer, while an intermediate rate of exchange between two conformers was observed for gAusIA^17^. In this paper, we present the co-crystal structure of gAusIA and rAusIA with *Ls*-AChBP that allowed us to understand the structural determinants underlying the equipotency at α7 nAChRs of two conformations. These studies revealed that the additional residue in the first loop of AusIA reduced structural rigidity and was a key contributor to globular and ribbon α-conotoxin equipotency.

## Results

### Co-crystal structures of gAusIA/*Ls*-AChBP and rAusIA/*Ls*-AChBP

*Ls*-AChBP was co-crystallized with synthesized gAusIA or rAusIA using the hanging drop method. The crystal structure of *Ls*-AChBP in complex with gAusIA and rAusIA were solved at 2.60 Å and 2.46 Å, respectively, using molecular replacement. Both belonged to the P3_1_21 space group with gAusIA/*Ls*-AChBP and rAusIA/*Ls*-AChBP having cell dimensions of a = 73.295 Å, b = 73.295 Å and c = 347.743 Å and a = 76.331 Å, b = 76.331 Å and c = 352.926 Å, respectively. Residues on flexible loops (mostly AChBP_loop F) were excluded from the final models where their electron density maps were ambiguous. The final structures were refined to an R_free_ value of 0.247 and 0.259 for gAusIA/*Ls*-AChBP and rAusIA/*Ls*-AChBP, respectively (Supplementary Table S1).

**Figure 1:**
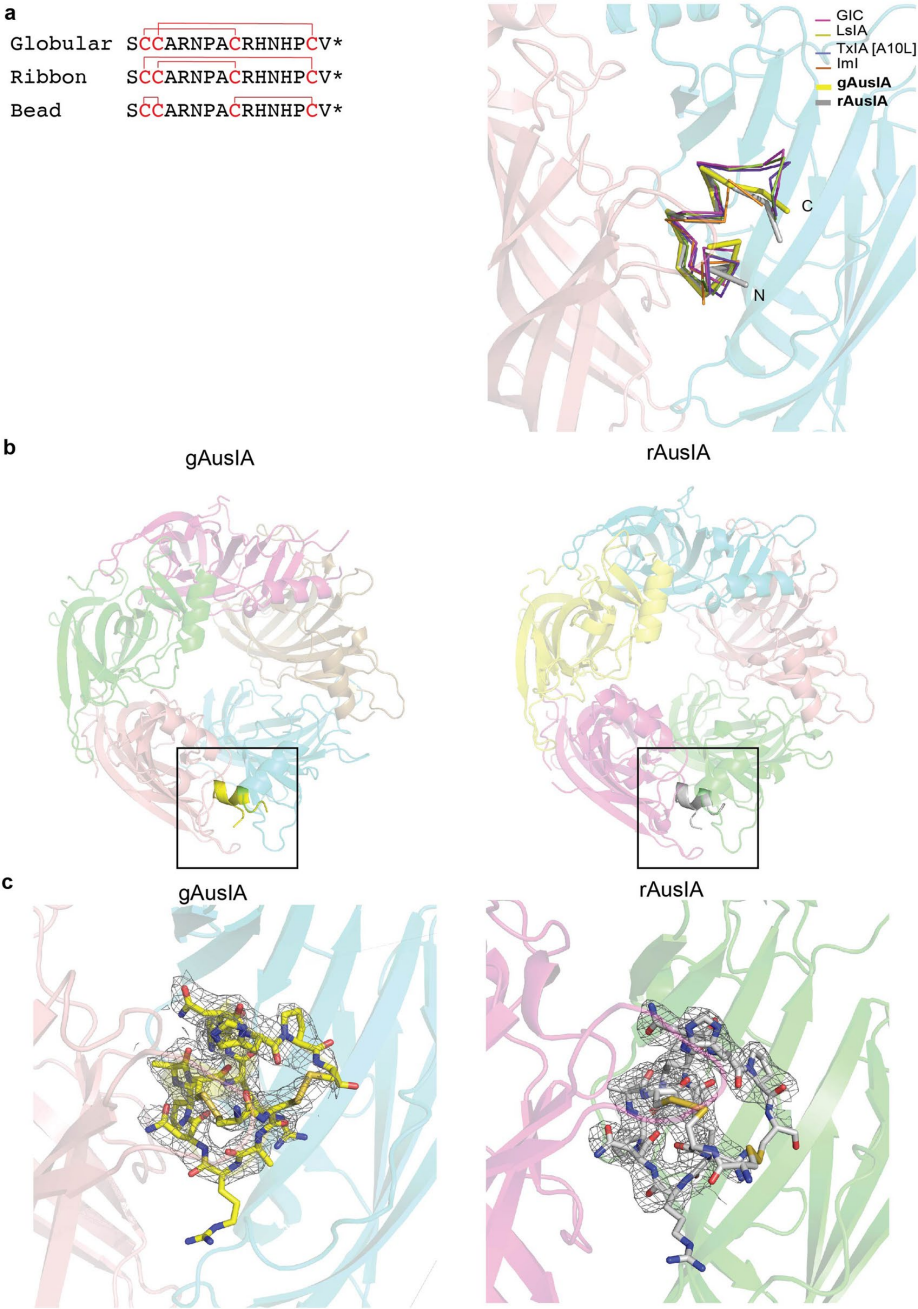
The gAusIA/*Ls*-AChBP and rAusIA/*Ls*-AChBP co-crystal structures. (**a**) The superimposition of gAusIA and rAusIA with previously co-crystallised α-conotoxins shows the similar backbone orientation. (**b**) Clear electron density for gAusIA and rAusIA is seen in only one binding pocket. (**c**) Fo-Fc maps for the ligand contoured to 1.0 s are shown. The typical binding mode of α-conotoxin is presented by both gAusIA and rAusIA in which the N and C termini of the bound α-conotoxin orienting towards the top and bottom of the ligand binding pocket respectively and the central helix abutting into the binding pocket.

The asymmetric unit contained one turbine-like pentamer and only one binding pocket was occupied by either gAusIA or rAusIA, possibly influenced by crystal packing (Fig. 1b,c). Indeed, more space is seen between the ligand from the pentamer and the adjacent crystal mate, as evidenced from the distance between Pro_189 with the closest residue on the C-loop of the adjacent crystal mate (7.9 Å for gAusIA and 9.1 Å rAusIA), consistent with the defined electron density for the ligand only being observed at this binding pocket (Supplementary Fig. S1a(i) and Fig. S1b(i)). Meanwhile, two binding interfaces are partially hindered by the adjacent crystal mate, as seen by distance between Pro_189 (Supplementary Fig. S1a(ii), b(ii)) or Ser186 (Supplementary Fig. S1a(iii), b(iii)) with the closest residue of the adjacent crystal mate, which is consistent with the weaker electron density in these binding pockets. The remaining binding interfaces show reduced ligand densities that could arise from an influence of crystal packing on AusIA pharmacology. The C-loop was displaced outward by 10.3 ± 0.34 Å in the occupied binding interface, which is comparable to previous co-crystal structures of bound α-conotoxins (based on the measurement between Cys187 C_α_ atom in the complex with HEPES/*Ls*-AChBP structure)^18^, while the C-loops of other binding interfaces remained in a closed conformation (Fig. 1b). The N-termini and C-termini of both gAusIA and rAusIA are highly solvent flexible and disordered, without clear electron density and were not fit into the structure. In contrast, a well-defined electron density for residues 6–14 is observed for both gAusIA and rAusIA (Supplementary Fig. S2). This core section of AusIA was used to guide the assembly of residues without clear electron density. While the electron density for both disulfide bonds of gAusIA is apparent, well-defined electron density is only presented for the Cys^II^-Cys^III^ disulfide bond of rAusIA (Supplementary Fig. S3). Despite this, a typical α-conotoxin binding orientation where the central helix protrudes into the ligand binding site of *Ls*-AChBP is exhibited by both gAusIA and rAusIA and superimposed with previously co-crystallised α-conotoxins (Fig. 1a).

### Structural interactions between gAusIA/rAusIA and *Ls*-AChBP

**Figure 2:**
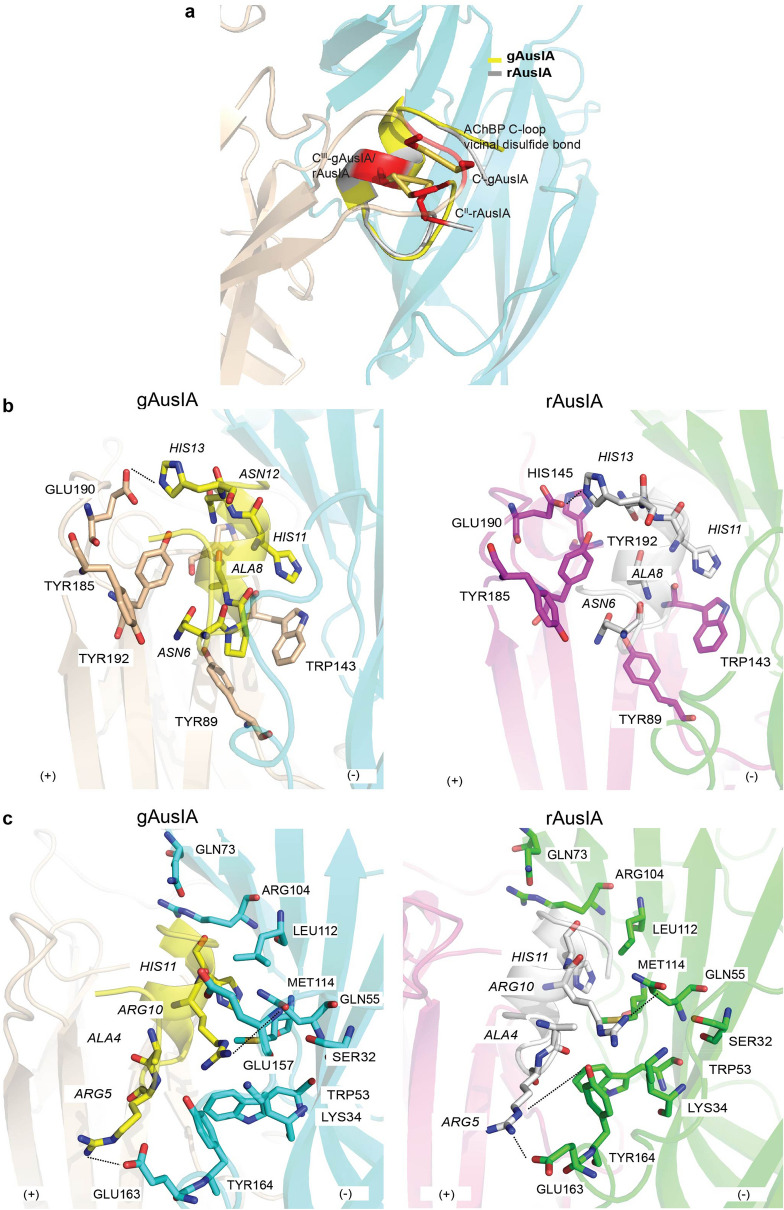
Binding interface between gAusIA/rAusIA with *Ls*-AChBP. **(a)** The stacking of disulfide bridges between Cys^I^-Cys^III^ of gAusIA and Cys^II^-Cys^III^ of rAusIA and Cys187-Cys188 of *Ls*-AChBP. gAusIA and rAusIA display similar pairwise interactions with *Ls*-AChBP. On the principal side (**b**), most contacts comprise conserved aromatic side-chain residues, particularly a hydrogen bond (dash line) formed between His13 and Glu190. On the complementary side (**c**), interactions are dominated by hydrogen bonds, specifically between Arg5 and Glu163, and Arg10 and Gln55. AusIA residues are in italics. Interaction distances are listed in Supplementary Table S2.

Superimposition of gAusIA and rAusIA co-crystal structures with *Ls*-AChBP revealed a comparable binding orientation in the binding pocket. In this orientation, the first disulfide bond (Cys^I^-Cys^III^ for gAusIA and Cys^II^-Cys^III^ for rAusIA) packs against the C-loop vicinal disulfide bond (Fig. 2a) with the central helix (residue 6–14) of both AusIA isomers protruding into the binding site in both complexes, forming an interacting core with the binding interface. Despite this overlap, the second disulfide bond of rAusIA undergoes larger conformational fluctuations compared to gAusIA, as suggested from the ambiguous electron density of the second disulfide bond in rAusIA (Supplementary Fig. S3). Despite this difference in rigidity, the extent and nature of the pair-wise interactions contributed by gAusIA and rAusIA are similar (Supplementary Table S2).

Both the principal and complementary faces contribute significantly to the interactions between gAusIA/rAusIA and *Ls*-AChBP. Most interactions on the principal side are between AusIA and the C-loop (Tyr185-Tyr192) of one of the *Ls*-AChBP protomers (Fig. 2b). Hydrophobic interactions were observed between the conserved aromatic side-chain residues Tyr89, Trp143, Tyr192, Tyr185 in AChBP and Asn6, Pro7, Ala8, His11, Asn12 and His13 of AusIA, with a salt bridge between His13 and Glu190. Meanwhile, the contacts between AusIA and β8–9 loop of *Ls*-AChBP on the complementary side involve hydrophobic, salt bridge and hydrogen bond interactions (Fig. 2c), including Ala4 interacting with Tyr164 via hydrophobic interactions, Arg5 of both gAusIA and rAusIA was built into the structure based on its backbone electron density and its geometry, allowing it to form a salt bridge with Glu163 and a hydrogen bond with Tyr164. Arg10 makes the most interactions on the complimentary face, being surrounded by residues Ser32, Lys34, Trp53, Gln55, Leu112, Met114, Glu157 and Tyr164. These interactions include hydrogen bonds between Arg10 and Gln55, and Glu157 and Tyr164. Finally, His11 resides in a pocket comprising charged Arg104, polar Gln73 and hydrophobic Leu112 (Supplementary Table S2).

### gAusIA and rAusIA interactions with the α7 nAChR

**Figure 3:**
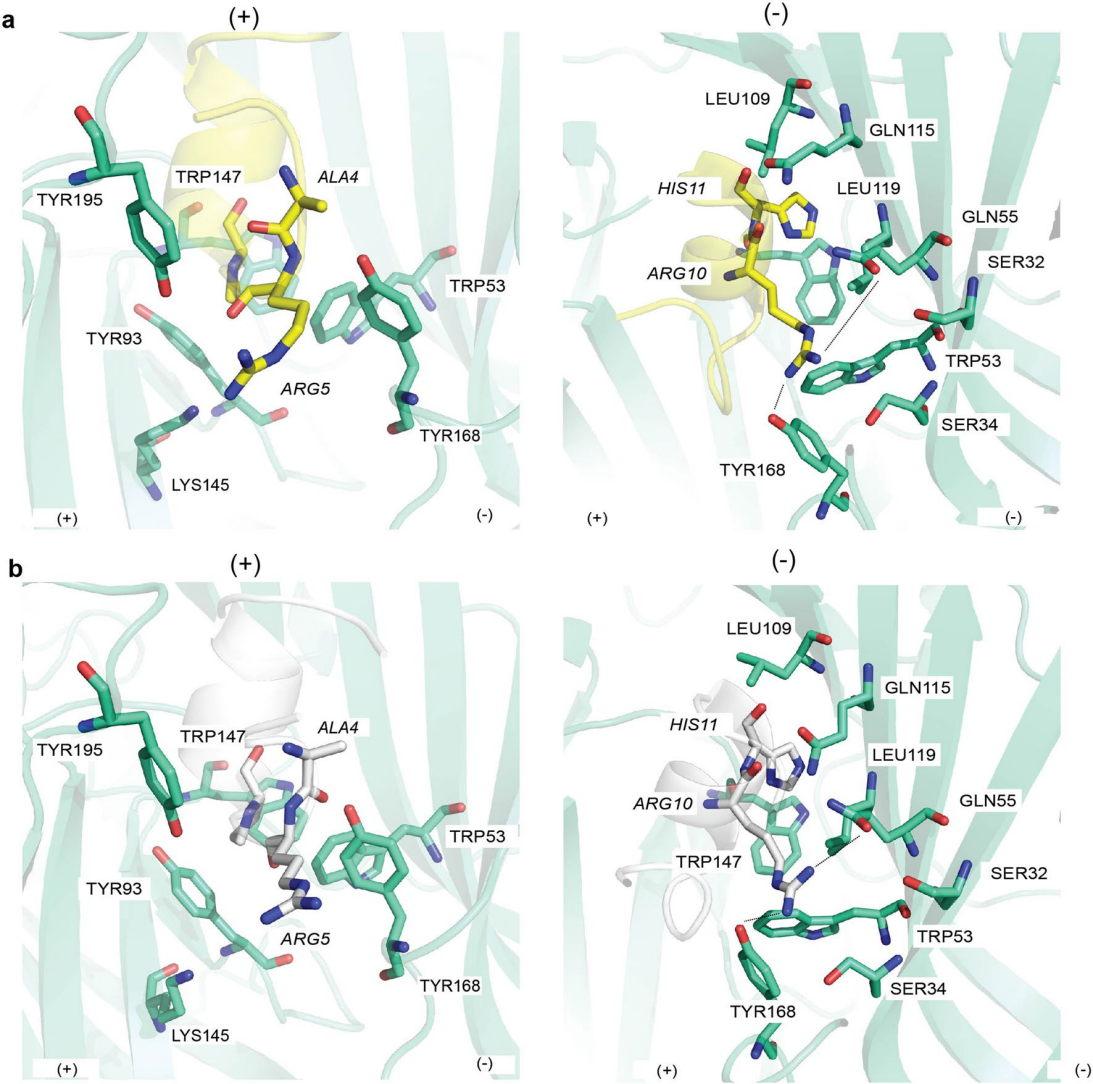
Major pairwise interactions between gAusIA (**a**) and rAusIA (**b**) at the binding interface formed by the principal (+) and complementary (-) face of human α7 nAChRs. gAusIA and rAusIA display similar binding interactions at human α7 nAChRs. On the principal face, Ala4 only forms hydrophobic interaction with α7_Tyr168 (*Ls*-AChBP_Tyr164), while a potential clash between Arg5 and α7_Lys155 could be made. Pro7 interacts mainly with aromatic side chain residues including Tyr93, Trp147 of the principal side and Trp55 of the complementary side. Arg10 makes the highest number of interactions on the complementary side, notably hydrogen bonds (dash lines) with α7_Gln55 and α7_Tyr166. The interacting surface of His11 consists of Trp147 on the principal side and Leu109, Gln117 and Leu119 on the complementary side of α7 nAChRs. AusIA residues are in italics. Interaction distances are listed in Supplementary Table S3.

The co-crystal structures of gAusIA and rAusIA/*Ls*-AChBP were used as a template to model the structure of α7 nAChR and to generate gAusIA and rAusIA/α7 homology model. The gAusIA/rAusIA contacting residues at α7 nAChR and *Ls*-AChBP share a high level of overlap in both position and side chain properties. However, a few notable differences were observed compared to *Ls*-AChBP. These include seeing His11 within a more hydrophobic pocket consisting of Gln117, Leu109 and Leu119 on α7 nAChR complementary side. In addition, Arg10 loses its hydrogen bonds with the complementary side due to the presence of α7_Thr77 and Leu109 in place of *Ls*-AChBP_Gln73 and Arg104, respectively (Fig. 3 and Supplementary Table S3).

Due to the equivalent pair-wise interactions within the binding pocket of α7 nAChRs made by gAusIA and rAusIA, it is expected that the contribution of different residues to the potency of gAusIA and rAusIA should be comparable. In order to validate these observations, selected residues at the terminals, 1st loop, and 2nd loop on both gAusIA and rAusIA were mutated (Supplementary Table S4). Typically, α-conotoxins have an N-terminal glycine and an amidated C-terminal Cys, while AusIA has a Ser14 replacing the conserved N-terminal Gly in typical α-conotoxins, and an extra C-terminal Val. In both co-crystal structures, both termini of AusIA presents with ambiguous electron density. To investigate the functional role of these unique termini, both isomers of AusIA with the substitution of Ser14 to the conserved Gly and the truncation of C-terminal Val were synthesized, AusIA [S1G] and AusIA [Δ16]. In addition, AusIA is an α5/5-conotoxin with either Ala4 or Arg5 being the likely additional residue inserted into the first loop. Ala4 forms hydrophobic interaction with *Ls*-AChBP_Tyr164 (α7_Tyr168), while an electrostatic clash appears between Arg5 and α7_Lys155 (Fig. 3). To examine the relevance of these residues to the activity of AusIA isomers, analogues with Ala4 or Arg5 deletions and Arg5Ala variants were prepared (AusIA [Δ4], AusIA [Δ5] and AusIA [R5A], respectively). Both Ala4 and Arg5 were also substituted with the conserved Ser typically found in the Ser-XXX-Pro motif of α-conotoxins (AusIA [A4S] and AusIA [R5S]). Pro7 is another highly conserved α-conotoxin residue that interacts with the conserved aromatic side chain residues in both nAChRs and AChBP orthosteric binding sites^19–21^. Similarly, at α7 nAChR the interacting surface of AusIA_Pro7 comprises of Tyr93 and Trp147 on the principal side and Trp55 on the complementary side. In the second loop, Arg10 makes the largest number of interactions on the complementary side of both *Ls*-AChBP and α7 nAChRs, notably through hydrogen bonds to α7_Gln55 and α7_Tyr166 (Fig. 3). To validate these observations, analogues where Pro7, Arg10 were replaced with Ala were synthesized (AusIA [P7A] and AusIA [R10A]). Finally, at α7 nAChRs the interacting surface of His11 is Trp147 on the principal side and Leu109, Gln117 and Leu119 on the complementary side (Fig. 3), providing important hydrophobic interactions for α-conotoxin inhibition at α7 nAChRs^20^. His11 was mutated to Leu with an aim of increasing AusIA potency at α7 nAChR by enhancing hydrophobic interactions.

### Truncation of loop 1 of AusIA restored typical antagonistic pharmacology to the globular and ribbon conformations

#### Conformational studies of AusIA analogues

The conformation of AusIA and its analogues were analyzed by CD spectroscopy (Supplementary Fig. S3). Both gAusIA and rAusIA showed poorly-defined α-helical structure with no evident local minima at ~ 205 nm and a positive band at ~ 187 nm. This is in contrast to the typical secondary structural characteristic of globular α-conotoxins but typical of the ribbon isomers. The CD data indeed agreed with previously reported NMR data where gAusIA and rAusIA displayed flexible structures with more than one uniquely folded structure identified^17^. Mutations to both gAusIA and rAusIA appeared to have no observable effect on conotoxin folding, with identical CD spectra for all mutants and wildtype AusIA.

#### Characterisation of gAusIA and rAusIA analogues at α7-containing nAChRs

**Figure 4:**
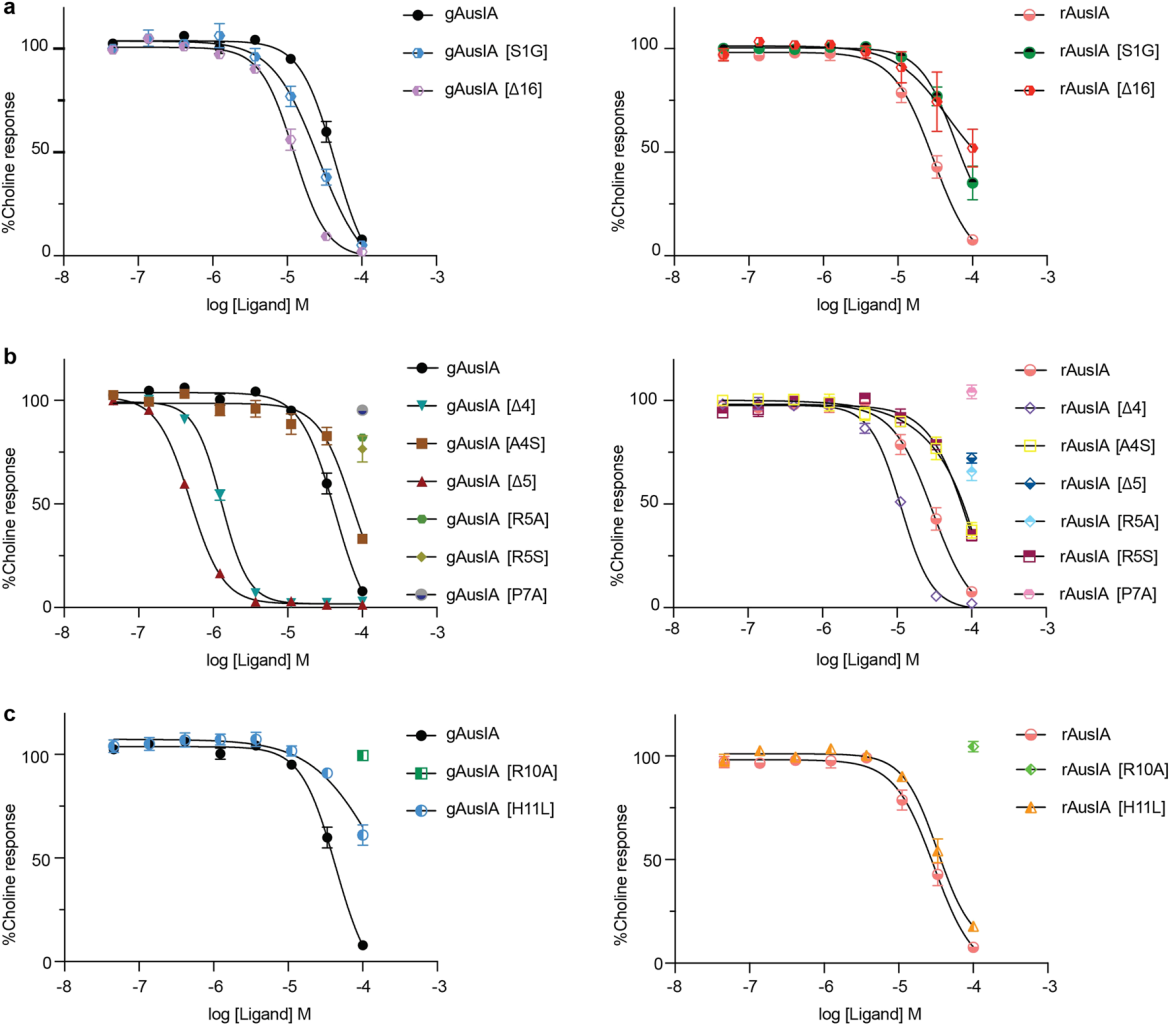
Functional characterisation of gAusIA and rAusAI at α7-containing nAChRs. Concentration response curves for gAusIA and rAusIA and their analogues at α7-containing nAChRs on SH-SY5Y cell are shown: (**a**) Termini changes, (**b**) loop 1 changes (**c**) loop 2 changes. Data represent mean $\pm$ SEM of triplicate data from three independent experiments.

Functional profiles of gAusIA and rAusIA were characterized on SH-SY5Y cells that endogenously express human α7-containing nAChRs. The C-terminal mutant gAusIA [S1G] showed a comparable potency to gAusIA, while rAusIA [S1G] lost two-fold potency compared to rAusIA. Deletion of Val16 at the N-terminus induced a four-fold increase in gAusIA potency but a 2.3-fold decrease in rAusIA potency (Fig. 4a and Table 1). Truncation of either Ala4 or Arg5 in loop 1 enhanced antagonistic activity of gAusIA by 47-fold and 80-fold relative to the wildtype AusIA, respectively. On the other hand, rAusIA [Δ4] increased two-fold potency, while rAusIA [Δ5] lost its antagonistic activity. Still, the removal of Arg5 resulted in a more than 100-fold difference in potency between gAusIA and rAusIA (Fig. 4b and Table 1), making AusIA [Δ5] behave more like a typical α-conotoxin where the globular is more potent than the ribbon conformation. Meanwhile, gAusIA [A4S] showed a comparable potency to gAusIA, while rAusIA [A4S] lost its activity. The A4S mutation showed no effect on gAusIA but induced a 1.5-fold loss in rAusIA potency. AusIA [R5A] and AusIA [R5S] lost activity, suggesting Arg5 in AusIA contribute pair-wise interactions in the binding pocket (Fig. 4b and Table 2). Finally, replacing Pro7 or Arg10 with Ala in the second loop produced a substantial loss in the antagonistic potency for both isomers (Fig. 4b and Table 1). On the other hand, replacing His11 with Leu abolished gAusIA inhibition, while rAusIA potency remained unchanged (Fig. 4c and Table 1).

**Table 1:** The IC_50_ for the inhibition of choline activation of α7-containing nAChRs in SH-SY5Y cells by AusIA and analogues.

	FLIPR SH-SY5Y			
	gAusIA		rAusIA	
	α7 nAChRs, IC_50_ ± SEM (μM)	Ratio^a^	α7 nAChRs, IC_50_ ± SEM (μM)	Ratio^a^
	47.0 ± 6.70	1.00	26.0 ± 5.00	1.00
S1G	33.0 ± 6.10	0.70	52.0 ± 5.60	2.00
∆16	12.0 ± 2.30	0.26	60.0 ± 9.90	2.30
∆4	1.0 ± 0.03	0.02*	11.0 ± 0.24	0.40
A4S	70.0 ± 12.90	1.49	69.0 ± 14.7	1.50
∆5	0.59 ± 0.08	0.01*	> 100	> 100
R5A	> 100	> 100	> 100	> 100
R5S	> 100	> 100	70.0 ± 10.0	2.70
P7A	> 100	> 100	> 100	> 100
R10A	> 100	> 100	> 100	> 100
H11L	> 100	> 100	30 ± 2.20	1.00

*Denotes significant change in IC_50_ compared to wildtype.

^a^IC_50_ ratio was calculated between gAusIA/rAusIA and its corresponding analogues.

### Structural comparisons of gAusIA and rAusIA to other α-conotoxins

**Figure 5:**
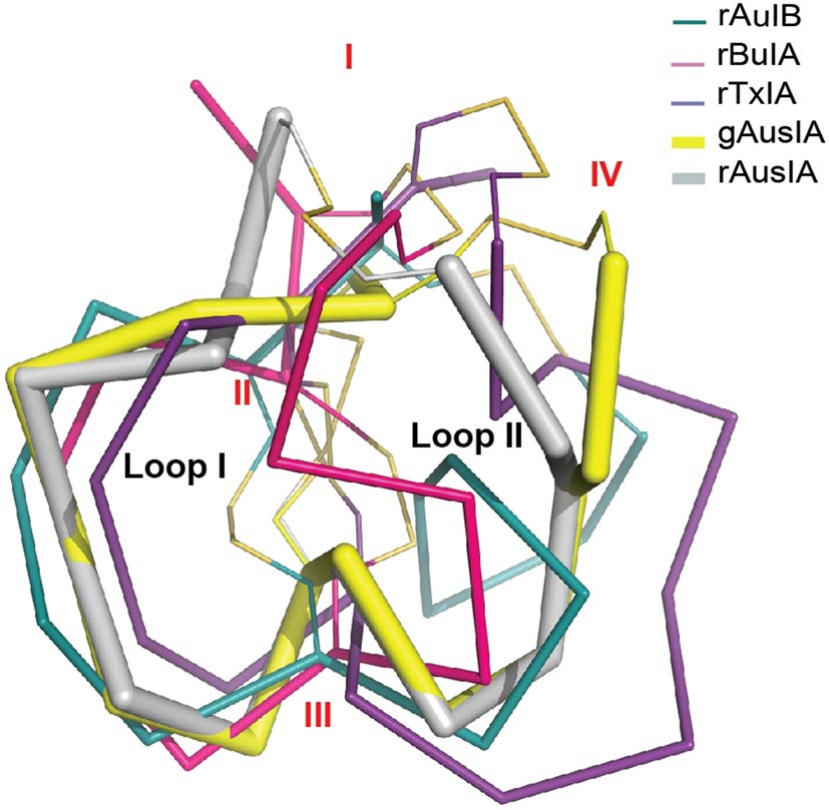
The superimposition of gAusIA and rAusIA with ribbon isomers of α4/4-BuIA (PDB 2NS3), α4/6-AuIB (PDB 1MXP) and α4/7-TxIA (PDB 6OTA).

Insights into the structural deviations between both AusIA isomers and other α-conotoxins were evaluated by structure superimpositions. Both isomers of AusIA overlayed well with other globular α-conotoxins to give backbone rmsds of 1.7–2.7 (Figs. 1a and 5). Although the presence of Ala4 expands the first loop and pushes Arg5 further towards the membrane face of the binding pocket, the overlay with globular α-conotoxins improved to 0.56–0.78 rmsds when the overlay was limited to the first loop. The structure of rAusIA was also compared with other published ribbon α-conotoxins, α4/4-BuIA (PDB 2NS3), α4/6-AuIB (PDB 1MXP) and α4/7-TxIA (PDB 6OTA). The ribbon isomer is generally characterized with a less well-defined structure than the globular isomer, with loop I being more rigid and loop II being more flexible due to a higher number of residues. rAusIA has a similar loop 1 conformation to other ribbon α-conotoxins. However, while loop 2 of ribbon BuIA, AuIB, and TxIA displayed larger structural variability reflected in their flexible conformations, rAusIA presented with a more compact loop 2 comparable to gAusIA and other globular α-conotoxins (Fig. 5).

To further explore the effect of extra residue in the first loop on AusIA antagonistic mechanism, a model of gAusIA [Δ5] was constructed and its binding mode evaluated by computational docking using an α7 nAChR homology model based on the co-crystal of gAusIA/*Ls*-AChBP. gAusIA [Δ5] model as expected has a smaller loop 1 that is equivalent to other typical globular α-conotoxins. A reasonable binding pose with a backbone conformation resembling that of the co-crystallized gAusIA was also presented by gAusIA [Δ5]. The pairwise interactions in gAusIA [Δ5]/α7 nAChR homology model were nearly identical to those observed in the co-crystal gAusIA/*Ls*-AChBP (Supplementary Fig. S5 and Supplementary Table S3).

### Amino acid insertions loop 1 of LsIA and TxIA [A10L] favour equipotency of globular and ribbon isomer

Deletion of Ala4 or Arg5 in AusIA favoured globular over ribbon isomer at human α7-containing nAChR subtypes. To determine if amino acid insertions in the first loop of α4/7-conotoxins resulting in the same effect, LsIA [∇A5] and TxIA [∇A5 A10L], also targeting human α7 nAChR, were assembled and both isomers generated. To mimic Arg5 of AusIA, LsIA [S5R] and LsIA [∇R6] were also synthesized in both folds, while TxIA [A10L] already contained Arg5 in its Ser-XXX-Pro motif. CD confirmed the structural integrity of chemically synthesised LsIA and TxIA [A10L] and their analogues (Supplementary Fig. S6), with globular LsIA and TxIA [A10L] having α-helical content, while the ribbon isomers had CD spectra indicative of a random coil conformation. Incorporating additional amino acids into the first loop had a profound effect on the globular conotoxin structure, with gLsIA [∇A5], gTxIA [∇A5 A10L] and gLsIA [∇R6] appearing less structured (Supplementary Fig. S6). In contrast, the ribbon mutants exhibited near identical CD spectra to the ribbon wildtype and no profound effect was observed for the LsIA isomers following the substitution of Ser5 into Arg.

**Figure 6:**
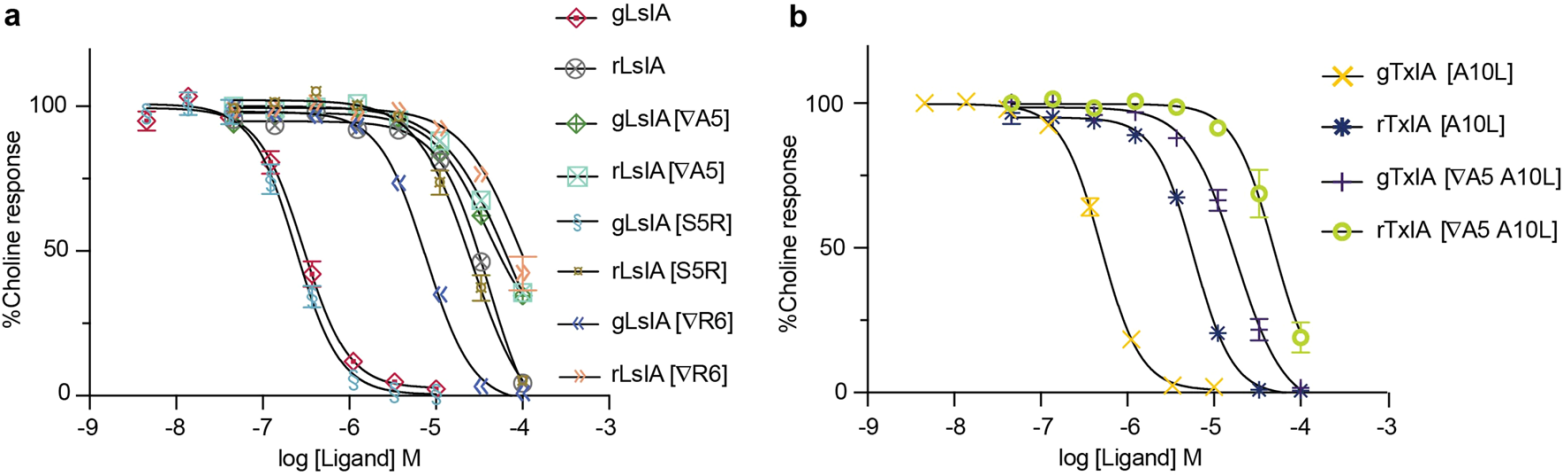
Functional characterisation of LsIA (**a**), TxIA [A10L] (**b**) and their analogues at human α7-containing nAChRs. Concentration response curves for LsIA, TxIA [A10L] and their analogues at α7-containing nAChRs on SH-SY5Y cells are shown. Data represent mean $\pm$ SEM of triplicate data from three independent experiments.

As expected, rLsIA and rTxIA [A10L] displayed no antagonistic activity at human α7-containing nAChRs. Interestingly, the addition of Ala in the first loop of both LsIA and TxIA [A10L] induced a similar phenomenon to that observed in AusIA, where the ribbon conformation showed a comparable potency to the globular isomer. gLsIA [∇A5] lost nearly 158-fold in potency relative to the wild type (48.0 ± 13.2 μM), while rLsIA [∇A5] had an IC_50_ of 57.0 ± 14.7 μM (Fig. 6a and Table 2). The IC_50_ of gTxIA [∇A5 A10L] was 36-fold lower than gTxIA [A10L], making the globular TxIA only twofold more potent than its respective ribbon conformation (Fig. 6b). The substitution of LsIA_Ser4 into Arg and the addition of Arg at position 6 of LsIA displayed differential effects on LsIA antagonism. The potency of gLsIA [S5R] remained the same as wildtype gLsIA, while the addition of Arg decreased gLsIA potency by 26-fold (IC_50_ of 8.0 ± 0.52 μM). However, the globular conformers of these mutants retained higher potency than their respective ribbon conformers.

**Table 2:** IC_50_ for the inhibition of choline activation of α7-containing nAChRs in SH-SY5Y cells by LsIA, TxIA [A10L] and analogues.

Peptides	Sequence	α7 nAChRs, IC_50_ ± SEM (μM)	Ratio^a^
gLsIA	SGCCSNPACRVNNPNIC	0.30 ± 0.063	1.00
rLsIA	SGCCSNPACRVNNPNIC	41.0 ± 3.4	1.00
gLsIA [∇A5]	SGCC**A**SNPACRVNNPNIC	48.0 ± 13.2	158.4*
rLsIA [∇A5]	SGCC**A**SNPACRVNNPNIC	57.0 ± 14.7	1.00
gLsIA [S5R]	SGCC**R**NPACRVNNPNIC	0.24 ± 0.040	1.00
rLsIA [S5R]	SGCC**R**NPACRVNNPNIC	27.0 ± 5.3	NA
gLsIA [∇R6]	SGCCS**R**NPACRVNNPNIC	8.0 ± 0.520	26.4*
rLsIA [∇R6]	SGCCS**R**NPACRVNNPNIC	66.0 ± 4.1	NA
gTxIA [A10L]	GCCSRPPCILNNPDLC	0.50 ± 0.036	1.00
rTxIA [A10L]	GCCSRPPCILNNPDLC	6.0 ± 0.123	1.00
gTxIA[∇A5 A10L]	GCCS**A**RPPCILNNPDLC	18.0 ± 4.0	36.0*
rTxIA[∇A5 A10L]	GCCS**A**RPPCILNNPDLC	39.0 ± 10.3	6.50

*Denotes significant change in IC_50_ compared to wildtype.

^a^IC_50_ ratio was calculated between by LsIA, TxIA [A10L] and its corresponding analogues.

### Double deletions enhance potency difference between gAusIA and rAusIA

**Figure 7:**
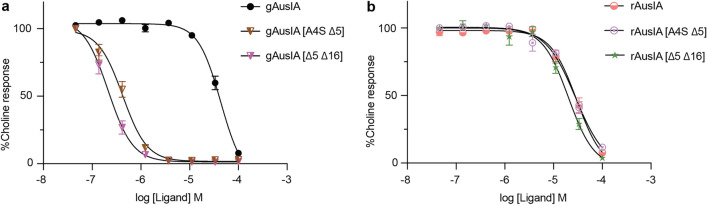
Functional characterisation of doubly-mutated gAusIA and rAusIA at human α7-containing nAChRs. Concentration response curves for gAusIA (**a**), rAusIA (**b)** and their analogues at α7-containing nAChRs on SH-SY5Y cells are shown. Data represent mean $\pm$ SEM of triplicate data from three independent experiments.

In order to gain further insight into the cumulative effects of these residues on AusIA inhibition at human α7-containing nAChRs, the double mutant AusIA [∆5 ∆16] was synthesised. AusIA [A4S ∆5] was also synthesized to examine whether it could stabilize the α-helical content of the peptide (Supplementary Table S4). Interestingly, both analogues displayed similar CD spectra to that of the wildtype (Supplementary Fig. S7), with gAusIA [∆5 ∆16] being 195-fold more potent than gAusIA, while the ribbon isomer potency was unaffected. This resulted in the ribbon isomer 112-fold less potent than the globular isomer. gAusIA [A4S ∆5] also showed a similar trend resulting in a globular isomer that was 67-fold more potent than ribbon isomer (Fig. 7 and Table 3).

**Table 3:** IC_50_ for the inhibition of choline activation of α7-containing nAChRs in SH-SY5Y cells by AusIA double mutant.

	FLIPR SH-SY5Y			
	gAusIA		rAusIA	
	α7 nAChRs, IC_50_ ± SEM (μM)	Ratio^a^	α7 nAChRs, IC_50_ ± SEM (μM)	Ratio^a^
	47.0 ± 6.70	1.00	26.0 ± 5.0	1.00
A4S	70.0 ± 12.90	1.49	32.0 ± 7.3	1.23
∆5	0.59 ± 0.080	0.01	51.0 ± 11.6	1.96
∆16	12.0 ± 2.30	0.26	60.0 ± 9.9	2.30
A4S ∆5	0.428 ± 0.112	0.009*	29.0 ± 1.1	1.00
∆5 ∆16	0.241 ± 0.062	0.005*	27.0 ± 5.3	1.00

*Denotes significant change in IC_50_ compared to wildtype.

^a^IC_50_ ratio was calculated between gAusIA/rAusIA and its corresponding analogues.

## Discussion

α-Conotoxin AusIA is equipotent in the native globular and ribbon conformations in contrast to most other α-conotoxins where the globular isomer is more potent^17^. To unravel the mechanisms underlying this unique feature of AusIA, we determined the pharmacology and co-crystal structures of AusIA globular and ribbon isomer with AChBP and docked these structures to a model of the α7 nAChR. These studies reveal that the first loop in ribbon and globular α-conotoxins plays a key structural role that influences α-conotoxin pharmacology at α7 nAChRs.

The pattern of pairwise interactions between gAusIA and rAusIA with *Ls*-AChBP and α7 nAChRs were similar, with most residues important for the globular isomer binding appearing equally important for ribbon isomer interactions. Confirmation of the structural basis underlying equipotency was confirmed by mutational analysis. In the co-crystal structure and α7 nAChR homology models of both AusIA isomers, Ala4 forms hydrophobic contacts with *Ls*-AChBP_Tyr164 (α7_Tyr168), while no electron density was seen for Arg5 side chain, suggesting it is not involved in any stabilizing contacts. The deletion of Ala4 and Arg5 increased the potency of gAusIA by 50-fold and 100-fold respectively, while rAusIA [∆4] had at least ten-fold higher potency than rAusIA [∆5] whose IC_50_ remained unchanged at the α7-containing nAChR. To the lesser extent, although no electron density was seen for both the terminals of both isomers, the deletion of the C-terminal Val caused a three-fold increase but a two-fold decrease to gAusIA and rAusIA potency, respectively. These results suggest that the equipotency shown by gAusIA and rAusIA at α7-containing nAChRs arise mainly from the insertion of an additional residue in the first loop and partly from residues in the termini.

To examine the structural effect of inserting an additional residue in the first loop brought to both AusIA isomers, structural imposition with α4/7-conotoxins was performed. Both AusIA isomers had backbones that overlayed well across the first and second loop, a feature not seen in previously reported ribbon α-conotoxins which had structures distinct from rAus1A (Fig. 5)^22,23^. gAusIA [∆5] when modelled at human α7 nAChRs displayed a more compact first loop equivalent to other typical globular α-conotoxins but otherwise retained pairwise interactions that matched gAusIA. Proton NMR of gAusIA showed Arg5 in solution was poorly defined (Supplementary Fig. S8a), supporting the weak α-helical content suggested by CD data and previous NMR studies showing gAusIA in solution exists in at least two conformations in intermediate exchange^17^. The structural instability of Arg5 also extended to the co-crystal structure, where its poorly defined electron density suggested Arg5 in the globular isomer might swing between interactions with Tyr164 and Glu163 in *Ls*-AChBP, or interact with Tyr168 on α7 nAChRs, which could explain the negative effect of the R5A or R5S mutations on α7 potency. Similarly Arg5 in rAusIA also shows a degree of structural flexibility (Supplementary Fig. S8b) and no well-defined secondary structural units^17^, allowing it to bind in a similar manner to the globular fold, explaining their equipotency. Previously, α-conotoxin BuIA also presented with similar NMR phenomenon as AusIA, in which the native globular disulfide connectivity of BuIA displayed multiple conformations in solution, whereas the non-native ribbon isomer displayed a single well-defined conformation. However, despite BuIA having multiple conformations in solution, one of the conformations of globular BuIA had a native α-conotoxin fold, which could be responsible for the high potency of globular BuIA at nAChRs^22,24^. Although gAusIA [∆5] presented with no well-defined α-helical secondary structure, the NMR spectra of gAusIA [∆5] was more defined compared to gAusIA, consistent with its enhanced potency at nAChRs (Supplementary Fig. S8c). Thus, it appears that the additional residue in the first loop of AusIA reduces structural rigidity in solution to adversely impact potency at α7-containing nAChRs.

Remarkably, insertion of Ala at the 5th position of LsIA and equivalent 4th position of TxIA [A10L] also made the globular isomers less active and equipotent to the ribbon isomer like AusIA. This decreased potency of the globular isomers could arise from the reduction in secondary structure evidenced from the loss in helical content of both gLsIA and gTxIA [A10L] observed by CD. In contrast, gLsIA [S5R] retained the potency relative to the wildtype and unchanged secondary structure, while insertion of Arg at position 6th of LsIA reduced helical content and potency 26-fold. The differential effect of inserting additional residue either before or within the Ser-X-Pro motif appears to be sequence specific, with the loop 1 sequence Ser-XXX-Pro rather than X-Ser-X-Pro having more prominent helical content and higher potency. In addition, removing a C-terminal residues in AusIA amplified this loop 1 effect, with the double mutant gAusIA [∆5 ∆16] having 195-fold higher potency and a 100-fold difference in the globular and ribbon isomer potencies. These results support earlier studies that suggested the C-termini contribute to differences in the binding mode between ribbon and globular AuIB^23^. However, the ribbon isomers of AuIB or TxIA [A10L] additionally had altered orientations and interactions within their second loop that also contributed to the differences in selectivity between globular and ribbon isomers^22,23^, rather than the first loop like AusIA.

In contrast to an expected enhancement of AusIA potency at α7-containing nAChRs, swapping His11 to Leu in the second loop to match PnIA [A10L] or TxIA [A10L]^20,25^ did not alter rAusIA [H11L] potency, while gAusIA [H11L] was inactive at α7-containing nAChRs. We suspect that replacing His11 with a hydrophobic residue might result in the loss of water-mediated hydrogen bonds between gAusIA_His11 and the backbone oxygen of α7_Leu119 (equivalent to *Ls*-AChBP_Met114) (Supplementary Fig. S9). Meanwhile, rAusIA contacts are closer to Leu109, Gln115 and Leu119 compared to gAusIA, allowing stronger hydrophobic interactions that might be expected to compensate for the loss of the water-mediated hydrogen bonding (Supplementary Table S3). On the other hand, results for the other mutations were consistent with our initial proposal of comparable contributions from Pro7 and Arg10 to the potency of gAusIA and rAusIA. The substitution of P7A and R10A in both gAusIA and rAusIA resulted in a complete loss of potency at α7-containing nAChRs. Pro7 is important in the stabilization of globular α-conotoxins in which the replacement of Pro with Ala typically causes a change of conformation that leads to a decrease or loss of α-conotoxins^23,26,27^. Although Pro7_AusIA appears less effective at stabilizing structure as evidenced by the ambiguous CD data, both isomers contribute identical extensive contacts with the conserved aromatic residues at the binding site. In addition, substitution of Arg10 with Ala in both isomers destabilised the interactions of both gAusIA and rAusIA with the complementary side, likely through the loss of key hydrogen bonds. Residue at this position (equivalent to residues at position 9 of other α-conotoxins) has previously identified as important for the modulation of the activity a range of globular α-conotoxins as well as ribbon AuIB^23,28–30^.

In conclusion, this study revealed that the binding mode of globular and ribbon isomers of AusIA at *Ls*-AChBP and α7 nAChRs were similar, explaining the equipotency of these isomers of α5/5-AusIA. It appears that insertion of an additional residue into the first loop of α-conotoxins reduces AusIA α-helical content, resulting in AusIA existing in multiple conformations in solution but binding to AChBP and α7-containing nAChRs in similar conformations for both the ribbon and globular folds. Given only one α-conotoxin belonging to α5/5 subclass has been identified to-date, we suggest it has evolved from more potent α-conotoxins with 4 residues in loop 1 to allow a broader range of nAChRs to be targeted. These insights provide a new understanding on α-conotoxins structure, allowing for a more systemic rational design of α-conotoxins as potential potent inhibitors with therapeutic leads.

## Methods

### Peptide synthesis by two-step oxidation

α-Conotoxin AusIA and its variants were assembled by solid-phase methodology on a Liberty PRIME peptide synthesizer (CEM, USA) using Fmoc chemistry and standard side chain protection, except for cysteine residues. Cys residues were orthogonally protected in pairs with acid-labile trityl (Trt) and acid-stable S-acetamidomethyl (Acm) respectively on Cys^I^-Cys^III^ and Cys^II^- Cys^IV^ for globular isomer, Cys^I^-Cys^IV^ and Cys^II^-Cys^III^ for ribbon isomer. Peptide cleavage from resin and global side chain deprotection were done by treatment with scavenger mixture (trifluoroacetic acid (TFA)/water/triisopropylsilane, 95:2.5:2.5, v/v/v) for 30 min at 40 °C on Razor system (CEM, USA). The cleaved peptides were precipitated with cold diethyl ether, centrifuged (5000 rpm × 3), redissolved in 50% aqueous acetonitrile (ACN) and lyophilized.

Disulfide bonds were formed selectively via a directed two-step oxidation. Trt protecting groups of the first Cys pairs were removed after peptide cleavage from resin, while Acm groups on the second Cys pairs remained intact. The oxidation of peptides was carried out in a buffer of 90% acetic acid (AcOH)/10% methanol (MeOH) with peptides at final concentration of 2 mg/mL. The first disulfide bridge formation between free cysteines was performed upon the dropwise addition of iodine (I_2_) (10 mg/mL dissolved in MeOH) while stirring until a pale yellow color persisted. The solution containing partially oxidized peptide was then diluted with an equal volume of 50 mM HCl in 50% aqueous MeOH. Simultaneous removal of the Acm group and the second disulfide bridge formation were accomplished by the continued addition of I_2_ (8 eq). The oxidation reaction was monitored via analytical HPLC (a linear gradient of 10–40% solvent B (90%ACN/0.05%TFA) over 30 min at a flow rate of 1 mL/min on a C18 column (Vydac 218TP, Grace) and electrospray ionization-mass spectrometry (ESI–MS)). The oxidation reaction was quenched by the addition of ascorbic acid and diluted 20-fold with solvent A. Bicyclic peptide was purified by RP-HPLC on a C18 Vydac column (Vydac 218TP, Grace) using a linear gradient of 5%–45% solvent B over 40 min at a flow rate of 16 mL/min. The final product was collected and analyzed by analytical HPLC and MALDI-TOF.

### AChBPs protein expression and purification

The over-expression of *Ls*-AChBP was performed as previously described^31^. Ubiquitin (Ub)-tagged AChBPs were used for radioligand binding assay. De-tagged *Ls*-AChBP was used for crystallization. Briefly, after immobilized metal affinity chromatography (IMAC) purification, *Ls*-AChBPs were removed from Ub-tag by DUB enzyme (produced in-house). De-tagged *Ls*-AChBP was further purified by size exclusion chromatography to assess homogeneity and oligomerization state. The IMAC purified *Ls*-AChBP was analyzed on a calibrated analytical HiLoad 16/600 column and (GE Health care) for *Ls*-AChBP respectively using AKTA fast protein liquid chromatography system (GE Health care). The fractions containing the proteins were pooled and concentrated to the desired concentration using an Amicon centrifuge filter (30-kDa cut-off, Millipore).

### Circular dichroism analysis

Circular dichroism (CD) analysis was used to study the secondary structure of peptides. CD spectra were obtained from Jasco J-810 spectropolarimeter (Jasco, Tokyo, Japan). Peptides were dissolved in 20 mM ammonium bicarbonate buffer pH 7.4 and 55% trifluoroethanol (TFE) at a final concentration of 50 μM. All measurements were done at room temperature under a nitrogen atmosphere (15 mL/min) with a scanning speed at 10 min and a 4 s response time. Absorbance was measured in the far-UV region (185–260 nm) via a cell with a path length of 1 cm and the capacity of 400 μL. Interference due to solvent, cell, or spectropolarimeter optics was eliminated via the subtraction of CD spectra of the pure solvents from those of the peptide. CD data in ellipticity was calculated to mean residue ellipticity ([θ]) using the equation: [θ] = θ/(10 × Np × (1000 × Np × C) × l), where θ is the ellipticity in millidegrees, C is the peptide molar concentration (M) of the peptide, l is the cell path length (cm), and Np is the number of peptide residues.

### Crystallization and data collection

Purified de-tagged *Ls-*AChBP and synthesized gAusIA/rAusIA were mixed at a molar ratio of 1:2 at 4 °C for 1 h before setting up crystallisation trials. Crystals were successfully grown at room temperature using the hanging drop method by mixing volumes of protein and reservoir solution at a ratio 2:1. The crystals for gAusIA/*Ls*-AChBP and rAusIA/*Ls*-AChBP were grown at 0.1 M calcium acetate hydrate, 18% PEG400, 0.1 M MES at pH 6.0 and 0.1 M calcium acetate hydrate, 12% PEG400, 0.1 M MES at pH 6.0, respectively. The crystals were cryo-protected with reservoir solution plus 20% (v/v) glycerol in liquid nitrogen.

### Structure determination and refinement

Diffraction data were collected at the MX2 beam line of Australian Synchrotron, Melbourne. Diffraction data were indexed, integrated via XDS and Molfsm and scaled via AIMLESS^32,33^. The structure was solved via molecular replacement using the PHASER^34^ crystallographic software with LsIA/*Ls*-AChBP (PDB 2C9T) as search model. Refinement against experimental data was done using Phenix.refine and COOT until clear electron densities for gAusIA and rAusIA were visible^35,36^. NCS restraints and TLS restrains were then applied and the final structures validated with MOLPROBITY and PDB validation^37^.

### Homology modelling

The homology modellings were performed via the project mode of the SWISSMODEL online server^38^. Briefly, the homology models were generated via the alignment of the ligand binding domain of the nAChRs with the crystal structure of the gAusIA/rAusIA with *Ls*-AChBP. The quality of alignment was manually adjusted. The resulting model was energy minimized using the GROMACS force filed in the program DEEPVIEW and models were analyzed in PyMol^38^.

### NMR structure characterization

NMR structure characterization was performed as previously described^39^. Briefly, peptide was dissolved in 500 μL of Milli-Q water (MilliPore, USA) and 50 μL of D_2_O (Cambridge isotopes). A Bruker 900 MHz Avance II spectrometer equipped with a cryoprobe (Bruker, Billerica, MA < USA) was used to acquire 1D ^1^H NMR spectra at 25 °C. Spectra were processed using TopSpin version 3.5 (Bruker). The chemical shift of water at 4.76 ppm was used as reference.

### Cell culture

Cell culture was performed as previously described^40^. Briefly, SH-SY5Y neuroblastoma cells (a gift from Victor Diaz, Max Plank Institute for Experimental Medicine, Goettingen, Germany) were cultured at 37 °C/5% (v/v) CO_2_ in RPMI media containing 2 mM L-glutamine and 15% (v/v) FBS. Cells were passaged every 3–5 days using 0.25% trypsin/EDTA at a dilution of 1:5. Experiments were conducted over several months and spanned on average a minimum of 10–20 passages. Responses were not affected by cell passage number with consistent control responses were recorded over the duration of experiments as responses^40^.

### FLIPR assay

FLIPR assay was performed as previously described^40^. Briefly, cultured SH-SY5Y cells were plated at a density of 100,000 cells per well on black-walled 384-well imaging plates and cultured for 48 h to form a confluent monolayer. Growth media was removed and incubated for 30 min at 37 °C with component A of the Calcium 4 assay kit. Intracellular increases in calcium in response to choline activating α7-containing nAChRs endogenously expressed by the SH-SY5Y cells that might include α7β2 nAChRs^41^. After incubation, the cells were transferred to the FLIPR (Molecular Devices). The changes in fluorescence correlated to intracellular calcium levels were measured using a cooled CCD camera with excitation 470–495 nm, emission 515–575 nm every 1 s. Camera gain and intensity were adjusted for each plate of cells yielding a minimum of 1500–2000 arbitrary fluorescence units (AFU) as a baseline fluorescence value. AusIA and analogues were added 10 min before applying choline for α7-containing nAChRs (30 μM). N-(5-Chloro-2,4-dimethoxyphenyl)-N′-(5-methyl-3-isoxazolyl)-urea (PNU-120596) is also used (10 μM) to measure activity at the α7-containing subtype on the FLIPR platform. The channel kinetics are too fast to measure otherwise. All compounds were diluted with physiological salt solution (PSS; 5.9 mM KCl, 1.5 mM MgCl_2_, 1.2 mM NaH_2_PO_4_, 5.0 mM NaHCO_3_, 140 mM NaCl, 11.5 mM glucose, 5 mM CaCl_2_, 10 mM HEPES, pH 7.4). FLIPR data was normalised to the maximum choline response in the SH-SY5Y cells to yield the %Fmax. A four-parameter Hill equation was fitted to the data using GraphPad Prism 9.0. Experiments were performed in triplicates in three independent experiments. IC_50_ values are reported as mean ± S.E.M. Comparison of the Log[IC_50_] values of each variant with the respective native α-conotoxin was carried out by pairwise comparison using an extra sum-of-squares F test with P < 0.05 in GraphPad Prism 9.0.

## Supplementary Information

Supplementary Information.

## Data Availability

Coordinates and structure factors for the gAusIA/*Ls*-AChBP and rAusIA/*Ls*-AChBP complexes have been deposited in the RCSB PDB with 7N0Y and 7N0W respectively.
