# Supplementary material for: Rigidity of loop 1 contributes to equipotency of globular and ribbon isomers of α-conotoxin AusIA
Source: Sci Rep. 2021 Nov 9;11:21928. doi: 10.1038/s41598-021-01277-4 (PMC8578332; doi:10.1038/s41598-021-01277-4)
Supplement: Supplementary file 1 — Supplementary Information. [file 41598_2021_1277_MOESM1_ESM.pdf]

Rigidity of loop 1 contributes to equipotency of globular and ribbon isomers of α-conotoxin AusIA

Thao NT Ho, Nikita Abraham and Richard J. Lewis*

Correspondence author:
Richard J. Lewis
Institute for Molecular Bioscience, The University of Queensland, Brisbane, 4067, Australia
Email: r.lewis@uq.edu.au

Supplementary information

Supplementary Table S1: Data collection and refinement statistic. Values in parentheses relate to the highest resolution shell

	***Ls*-AChBP-gAusIA**	***Ls*-AChBP-rAusIA**
**Data collection**		
Space group	$P3_121$	$P3_121$
Cell dimensions, Å	a=73.3 Å, b=73.3 Å, c=347.7 Å	a=76.3 Å, b=76.3 Å, c=352.9 Å
Cell dimensions, °	a=90 °, b=90 °, g=120.00 °	a=90°, b=90°, g=120°
Resolution, Å	46.88–2.58 (2.69–2.58)	48.25–2.46 (2.55–2.46)
Rsym	0.071 (0.55)	0.076 (1.08)
I/Is	20.8 (3.0)	19.5 (2.2)
Completeness (%)	99.8 (98.8)	99.2 (97.4)
Multiplicity	19.8 (20.6)	20.1 (20.4)
Total no. of reflections	703869 (86982)	891944 (91089)
Unique reflections	35545 (4231)	44280 (4459)
**Refinement**		
Resolution Å	46.88–2.58	48.25–2.46
Rwork/Rfree	0.22/0.26	0.21/0.25
rmsd bond distance, Å	0.01	0.01
rmsd bond distance, Å	1.3	1.3
Average B-factor	70.0	70.0

Supplementary Table S2: Receptor-ligand interactions observed in the gAusIA and rAusIA co-crystal structures with *Ls*-AChBP.

	**Principal (+)**	**Distance (Å)**		**Complementary (-)**	**Distance (Å)**	
		**gAusIA**	**rAusIA**		**gAusIA**	**rAusIA**
Cys 2						
Cys 3	Tyr185<br>Cys187<br>Cys188	>5<br>3.9<br>5.0	4.3<br>3.8<br>4.9			
Ala 4				Tyr164	3.3	3.1
Arg 5				Glu163<br>Tyr164	3.9<br>3.5	3.5<br>3.2
Asn 6	Tyr 185<br>Tyr 192<br>Asp194	4.9<br>4.2<br>>5.0	4.6<br>5.0<br>4.5			
Pro 7	Tyr89<br>Trp143	3.8<br>3.6	4.3<br>3.8	Trp53<br>Met114	4.6<br>4.9	4.4<br>4.2
Ala 8	Ser142<br>Thr144<br>Tyr192	5.0<br>4.0<br>5.0	5.0<br>4.5<br>5.0			
Cys 9	Tyr185<br>Tyr192	5.0<br>4.4	>5.0<br>3.8			
Arg 10				Ser32<br>Lys34<br>Trp53<br>Gln55<br>Glu157<br>Tyr164	>5.0<br>3.7<br>3.1<br>3.3<br>>5.0<br>2.6	4.4<br>3.5<br>5.5<br>>5.0<br>2.1<br>2.6
His 11	Trp143<br>Thr144	4.4<br>3.1	3.8<br>3.6	Arg104<br>Leu112<br>Met114	4.2<br>4.6<br>3.7	4.1<br>3.5<br>3.3
Ans 12	His146<br>Glu149<br>Tyr192	4.1<br>4.5<br>2.9	3.1<br>4.7<br>4.6	Gln73<br>Arg104	4.1<br>3.7	2.9<br>2.7
His 13	Cys188<br>Pro189<br>Glu190<br>Tyr192	3.8<br>5.0<br>3.6<br>4.0	3.2<br>>5.0<br>2.8<br>3.6			
Pro 14				Gln55<br>Thr57<br>Leu112	4.5<br>5.0<br>5.0	3.8<br>4.5<br>3.2
Cys 15				Glu157	4.3	3.5

Supplementary Table S3. Major receptor-ligand interactions observed in the gAusIA and rAusIA docking to α7 nAChRs

	Principal (+)	Distance (Å)		Complementary (-)	Distance (Å)	
		gAusIA	rAusIA		gAusIA	rAusIA
Ala 4				Tyr168	3.1	3.1
Arg 5	Lys155	5.0	>5.0	Tyr168	3.9	4.5
Pro 7	Tyr93<br>Trp147	3.1<br>3.6	3.5<br>3.3	Trp53	4.7	5.0
Arg 10				Trp53<br>Gln55<br>Tyr168	 4.7<br>2.4	4.6<br>3.6<br>3.0
His 11	Trp147	3.3	3.8	Leu109<br>Gln115<br>Leu119	3.4<br>5.0<br>4.8	2.7<br>5.0<br>3.6

Supplementary Table S4. Amino acid sequences of gAusIA, rAusIA and variants

Peptide name	Sequence	Theoretical mass	Observed mass
AusIA	SCCARNPACRHNHPCV	1763.06	1763.12
AusIA [S1G]	**G**CCARNPACRHNHPCV	1734.04	1734.03
AusIA [Δ16]	SCCARNPACRHNHPC	1663.93	1663.60
AusIA [Δ4]	SCCRNPACRHNHPCV	1691.99	1691.34
AusIA [A4S]	SCC**S**RNPACRHNHPCV	1779.06	1779.15
AusIA [Δ5]	SCCANPACRHNHPCV	1606.88	1606.66
AusIA [R5S]	SCCA**S**NPACRHNHPCV	1693.95	1693.43
AusIA [R5A]	SCCA**A**NPACRHNHPCV	1677.96	1677.34
AusIA [P7A]	SCCARN**A**ACRHNHPCV	1737.03	1737.45
AusIA [R10A]	SCCARNPAC**A**HNHPCV	1677.96	1677.30
AusIA [H11L]	SCCARNPACR**L**NHPCV	1739.08	1739.23
AusIA [A4S Δ5]	SCC**S**NPACRHNHPCV	1622.88	1622.57
AusIA [Δ5 Δ516]	SCCANPACRHNHPC	1507.75	1507.34

Supplementary figure legends

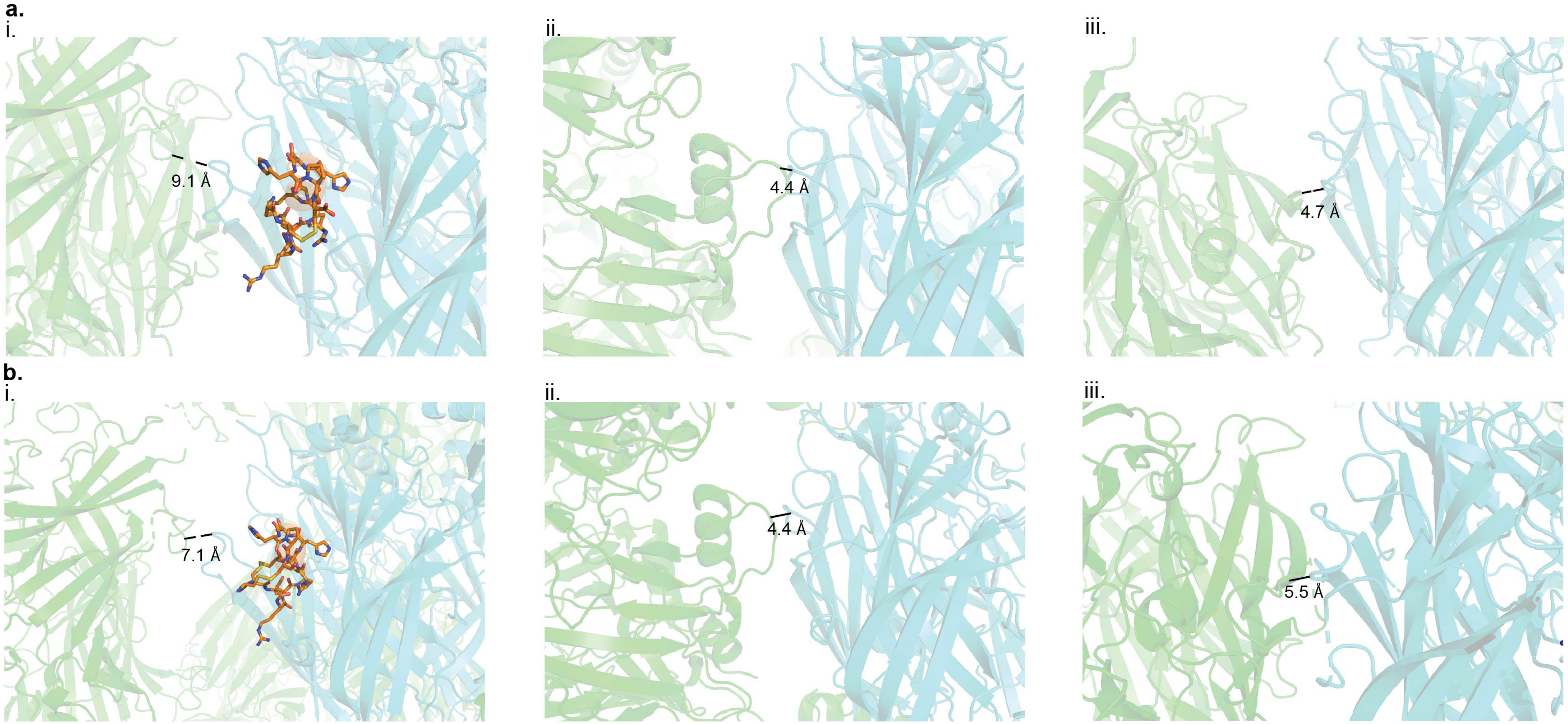

Figure S1. The effects of crystal packing on gAusIA (a) and rAusIA (b) at the binding interface of *Ls*-AChBP. More space is seen between the ligand from the pentamer and the adjacent crystal mate, consistent with the defined electron density for the ligand only being observed at this binding pocket (i). Meanwhile, the other binding interfaces are either partially hindered by the adjacent crystal mate (ii and iii), consistent with the weaker electron density in these binding pocket.

a. gAusIA **b.** rAusIA

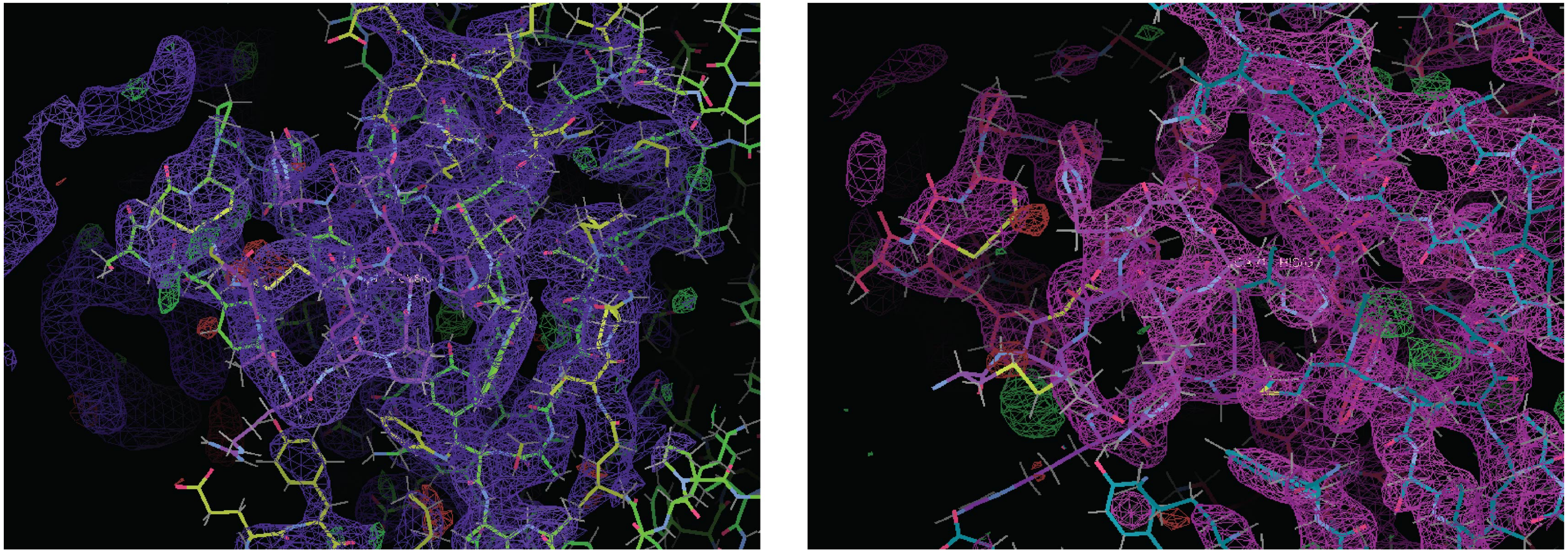

Figure S2. Clear electron density in the binding pocket is present for residues from residue 6 to residue 14 of both gAusIA (a) and rAusIA (b). Fo-Fc maps for the ligand contoured to 1.0σ are shown

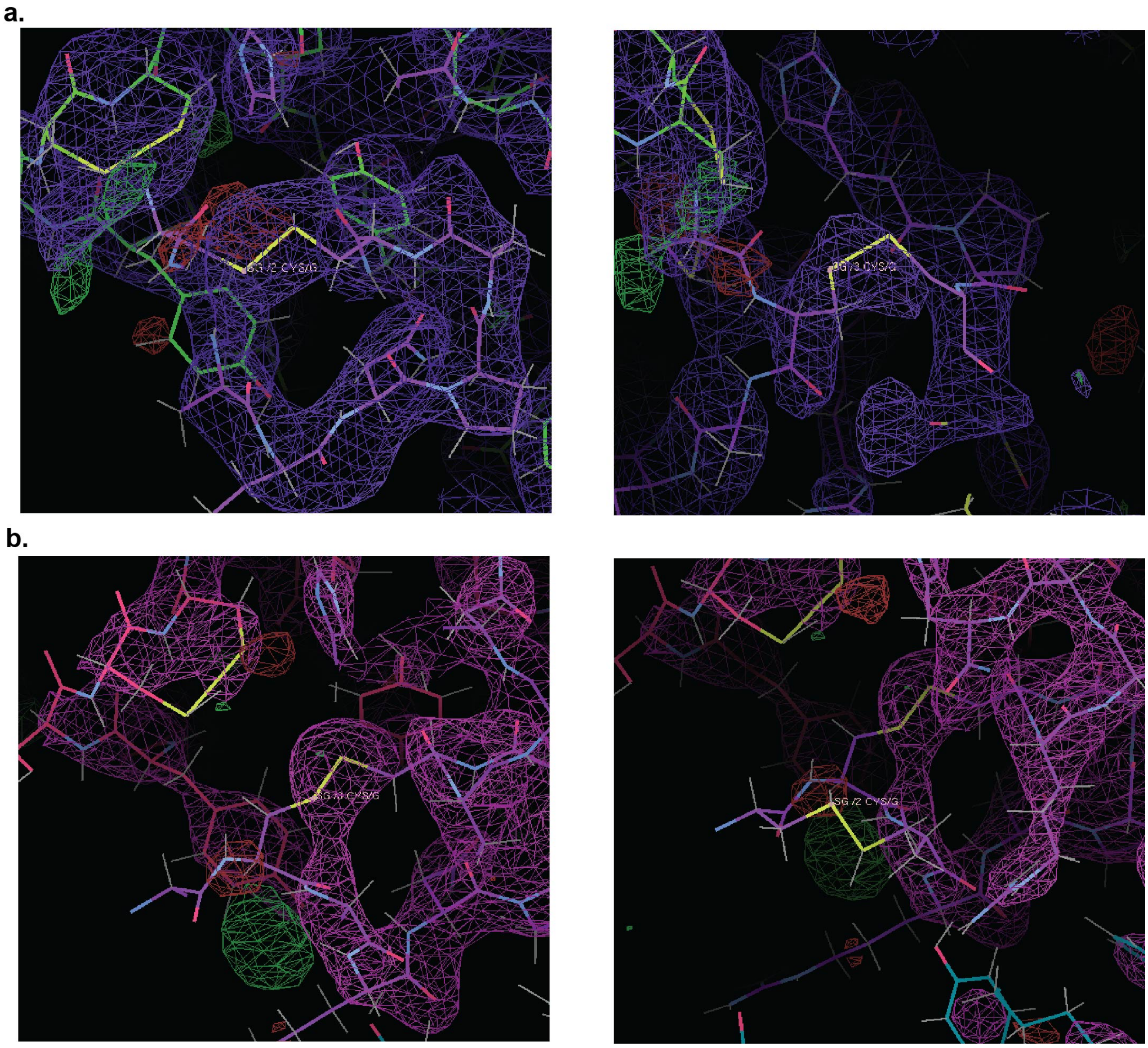

Figure S3. Electron density for the disulfide bonds of gAusIA (a) and rAusIA (b). Clear electron density is seen for both disulfide bonds of gAusIA. Meanwhile, only the first disulfide bond of rAusIA (CysI^{I-}Cys^{III}) (left panel) is present, while there is no electron density for the second disulfide bond (Cys^{I}-Cys^{IV}) (right panel). Fo-Fc maps for the ligand contoured to 1.0σ are shown

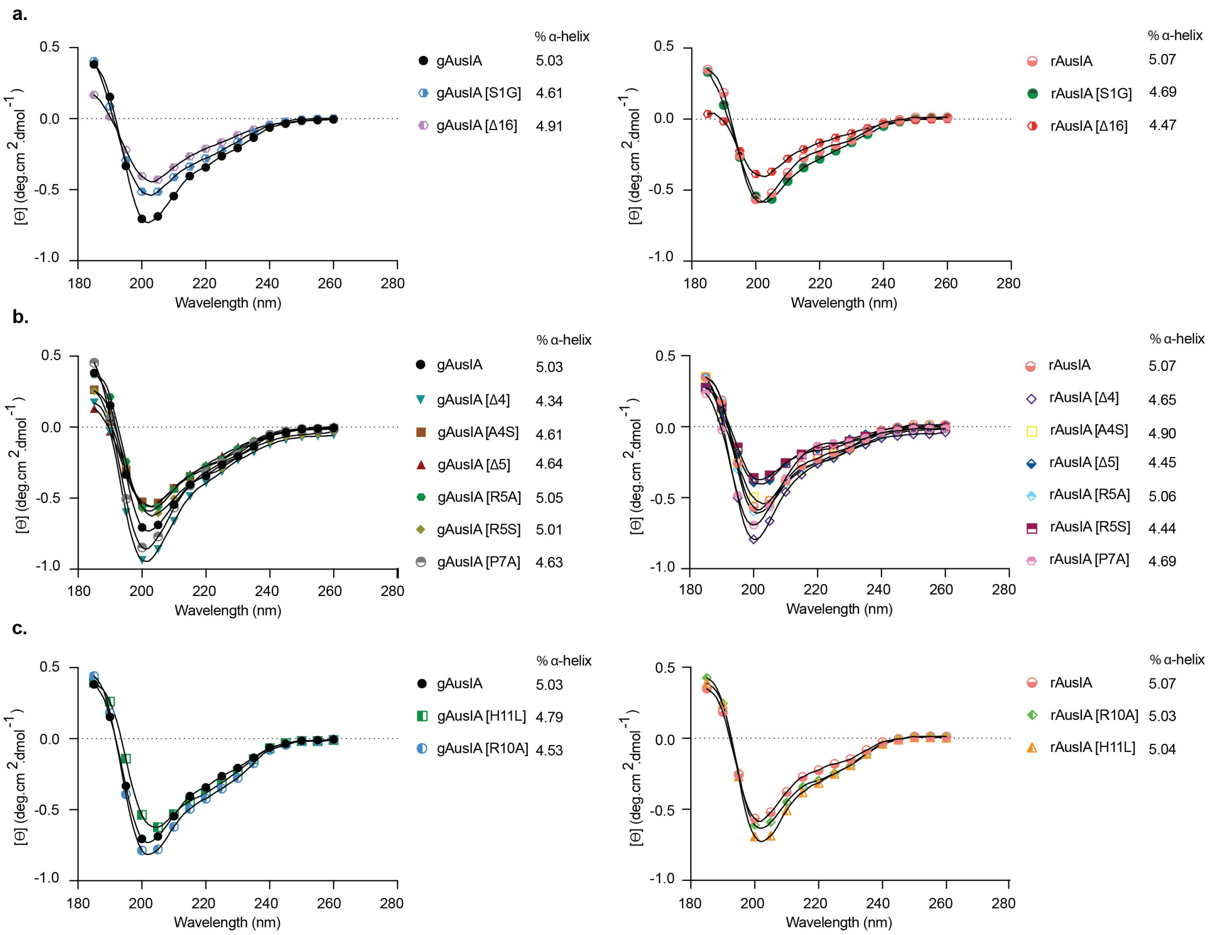

Figure S4. CD spectra of gAusIA and rAusIA and their analogues with mutation at both termini (**a**), in the first loop (**b**) and in the second loop. The%helix is indicated in the legend and was estimated via K2D3 [2].

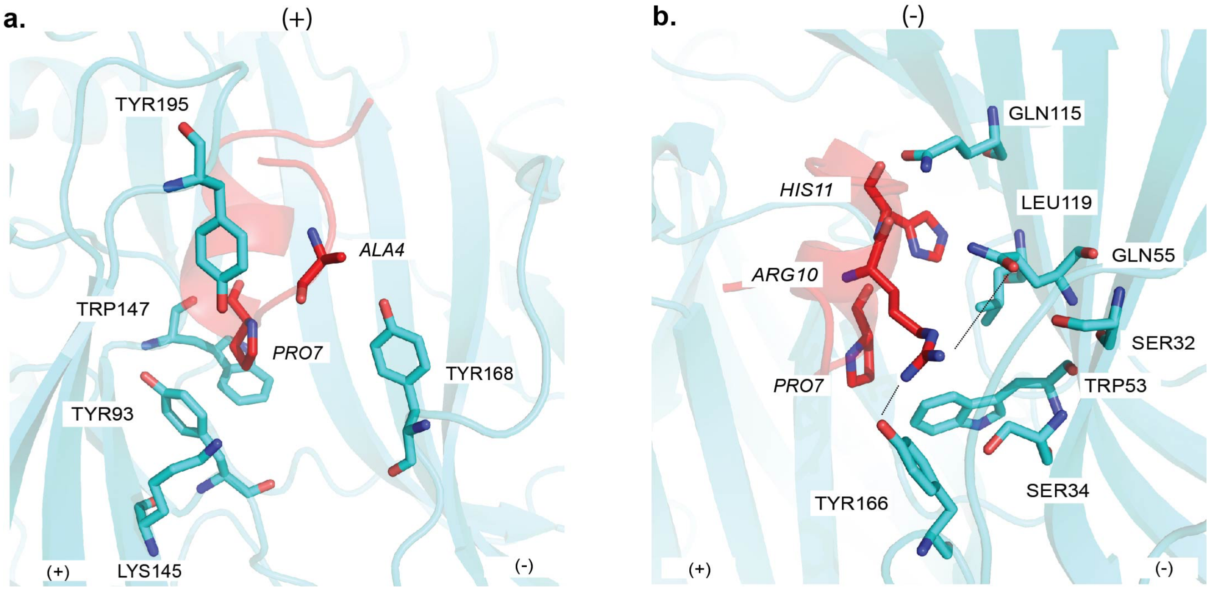

Figure S5. Notable interactions between model gAusIA [Δ5] at the binding interface formed by the principal (a) and complementary face (b) of human α7 nAChRs.

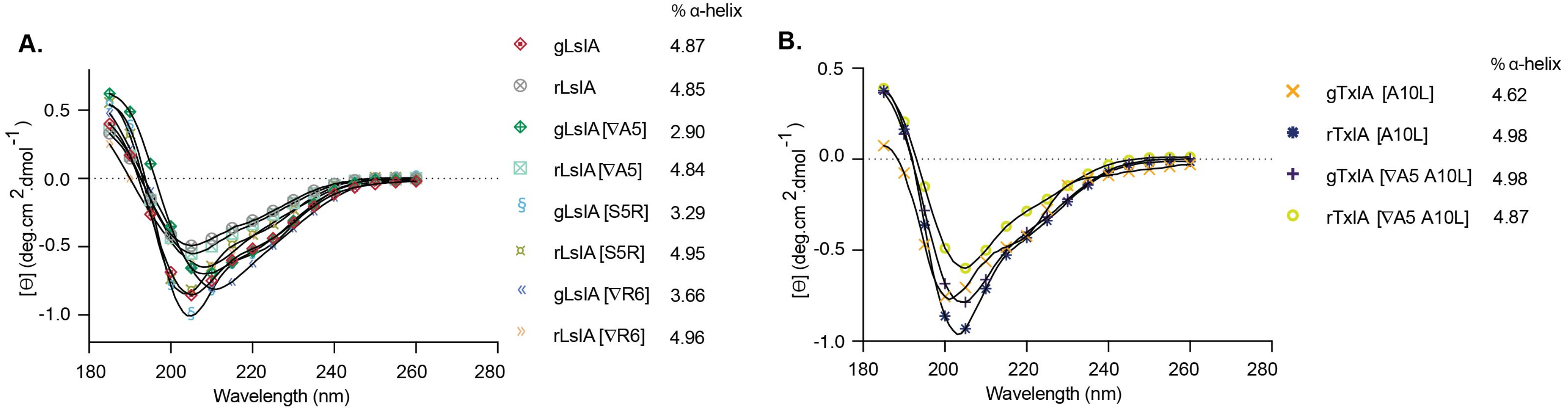

Figure S6. CD spectra of LsIA (a), TxIA [A10L] (b) and their analogues. The%helix is indicated in the legend and was estimated via K2D3 [1]

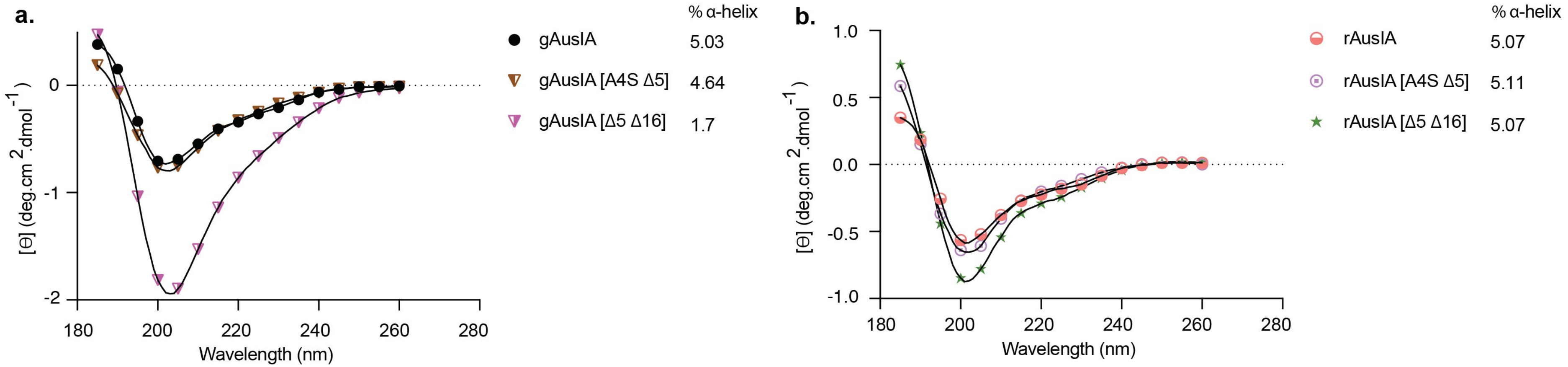

Figure S7. CD spectra of doubly-mutated gAusIA (A) and rAusIA (B) analogues. The%helix is indicated in the legend and was estimated via K2D3 [1]

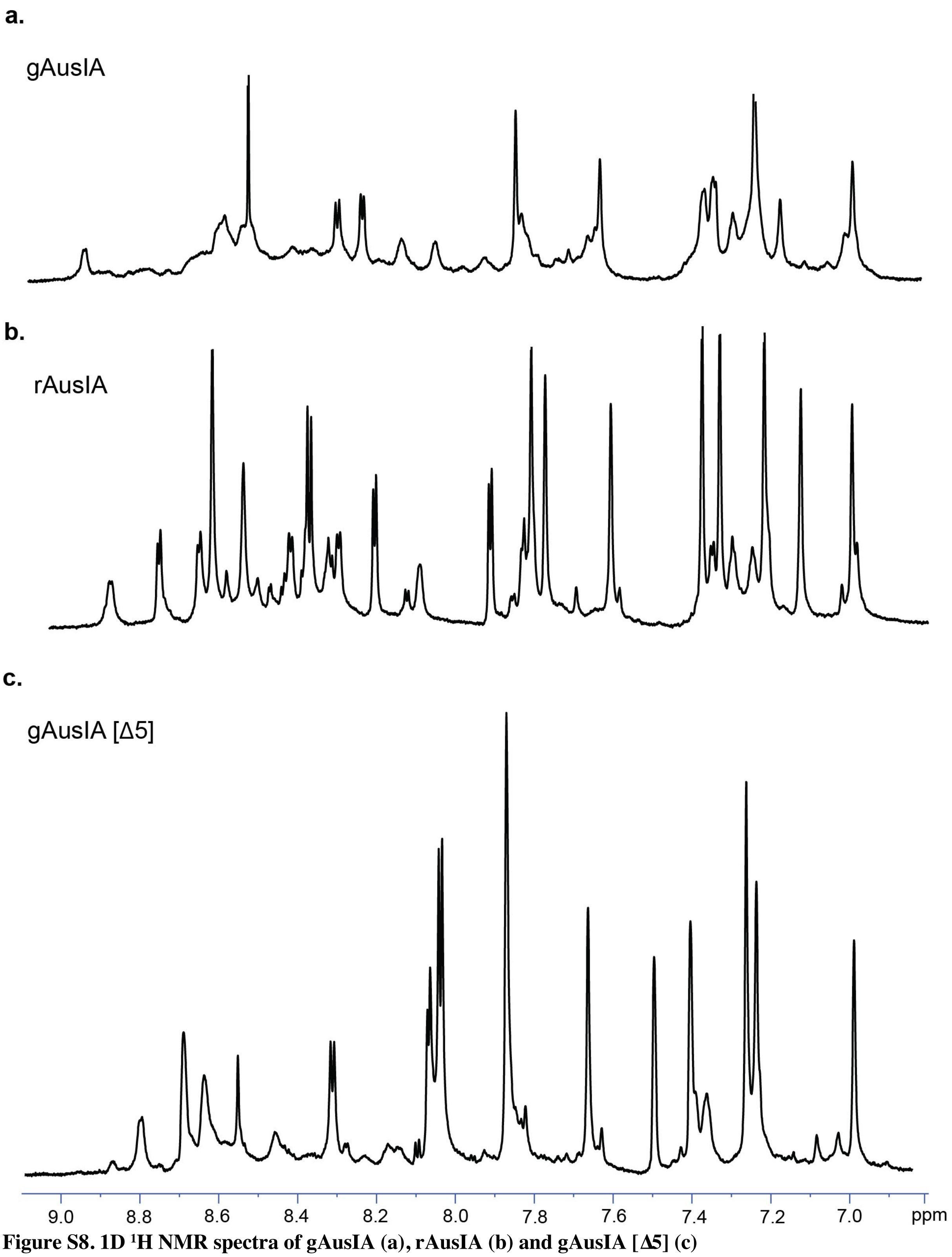

Figure S8. 1D ^{1}H NMR spectra of gAusIA (a), rAusIA (b) and gAusIA [Δ5] (c)

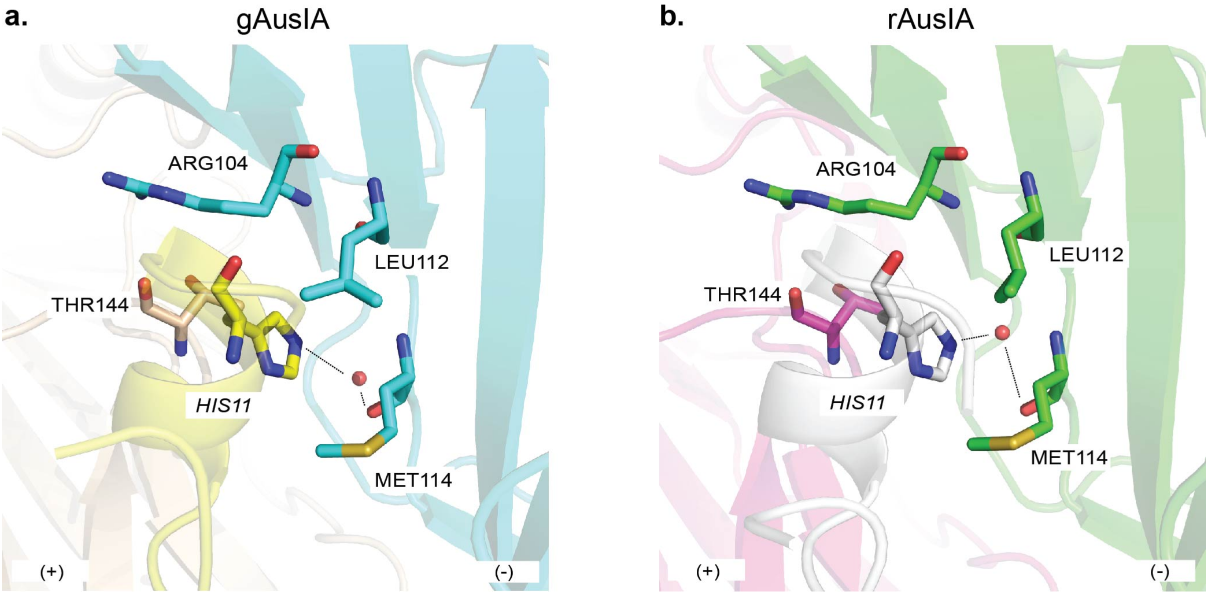

Figure S9. Water-mediated hydrogen bond is seen between His11 and the backbone oxygen of Met114 on the complementary side of *Ls*-AChBP in both the co-crystal structure of gAusIA (a) and rAusIA (B) with *Ls*-AChBP

1 Louis-Jeune, C., Andrade-Navarro, M. A. & Perez-Iratxeta, C. Prediction of protein secondary structure from circular dichroism using theoretically derived spectra. *Proteins* **80**, 374-381, doi:10.1002/prot.23188 (2012).
